# Dual plasmonic modes in the visible light region in rectangular wave-shaped surface relief plasmonic gratings

**DOI:** 10.1038/s41598-023-30083-3

**Published:** 2023-03-31

**Authors:** Rahmat Hidayat, Jalu Setiya Pradana, Alvin Fariz, Susi Komalasari, Siti Chalimah, Herman Bahar

**Affiliations:** 1 Physics of Magnetism and Photonics Research Division, Physics Study Program, Faculty of Mathematics and Natural Sciences, Institut Teknologi Bandung, Jl. Ganesha 10, Bandung, 40132 West Java Indonesia; 2 Present Address: Department of Biosystems, Biosensors Group, KU Leuven, 3001, Leuven, Belgium

**Keywords:** Nanophotonics and plasmonics, Nanophotonics and plasmonics, Sub-wavelength optics, Optical sensors

## Abstract

Rectangular wave-shaped surface-relief plasmonic gratings (RSR-PGs) have been fabricated from a hybrid polymer by employing a simple nanoimprint photocuring lithography technique using a silicon template, followed by gold nanolayer metallization on top of the formed replica structure. By forming a one-dimensional (1D) plasmonic grating with a periodicity of approximately 700 nm, a reflectance spectral dip was experimentally observed in the visible light region, from 600 to 700 nm, with increasing incident angle from 45° to 60°. This dip can be associated with surface plasmon resonance (SPR) wave excitation, which is coupled with the diffraction order *m* =  − 2. The calculations of reflectance spectra simulation using the rigorous coupled wave analysis (RCWA) method have also been carried out, resulting in the appearance of an SPR dip in the range of 600–700 nm, for incident angles in the range of 45°–65°, which agrees with the experimental results. Interestingly, these RSR-PGs show richer plasmon characteristics than the sine-wave-shaped plasmonic gratings. The experimental and spectral simulation results revealed two different plasmonic excitation modes: long-range SPR and quasi-localized SPR (LSPR). While the long-range SPR was formed above the ridge sections along the grating structure surface, the quasi-localized SPR was locally formed inside the groove. In addition, for RSR-PGs with a narrow groove section, the long-range SPR seems to be coupled with the periodic structure of the grating, resulting in the appearance of plasmonic lattice surface resonance (LSR) that is indicated by a narrower plasmon resonance dip. These characteristics are quite different from those found in the sine wave-shaped plasmonic gratings. The present results may thus provide better insights for understanding the plasmon excitations in this type of rectangular plasmonic grating and might be useful for designing their structure for certain practical applications.

## Introduction

Surface plasmon resonances (SPR) have been widely applied beyond just as refractive index sensors, expanding for broader applications such as the detection of biochemical substances, such as proteins, DNA, viruses, bacteria, toxic chemicals, pesticides etc. in rapid medical diagnosis and environmental monitoring^1–4^. From a scientific point of view, it is interesting to further explore SPR to study and manipulate light-matter interactions in the subwavelength regime, especially in the near-field regime and beyond the diffraction limit regime. SPR, also referred to as surface plasmon polariton (SPP), is a collective oscillation of electrons on a metal surface due to the resonance phenomenon between incident electromagnetic waves (light) and electrons at the metal–dielectric interface. In order to generate the resonance condition, SPR must be generated using a coupling element, such as prisms and gratings, or a functional structure, such as a waveguide, fiber optics, or other unique nanostructures^5–9^. The conventional SPR system uses a prism coupler configuration, commonly known as the Kretschmann or Otto configurations^1–3,10^. However, these configurations require complex optical and instrumentation systems. In many commercially available systems, the system is only able to measure the reflection intensity vs. the incident light angle, while the information on the SPR dip shift is sometimes important. In contrast, the SPR system that uses a grating (hence a plasmonic grating) as the coupling element requires a less complicated optical and instrumentation system. A different approach to generating plasmons is to use metal nanoparticles, which take advantage of localized SPR (LSPR) properties that are independent of the incident angle. However, metal nanoparticles, particularly gold nanoparticles, are quite expensive because their production requires a high-precision synthesis process. In addition, metal nanoparticles also have a limited shelf life. Therefore, the use of metal nanoparticles is still limited for highly sensitive sensing/detection purposes with a limited quantity of sample/analyte, such as for the detection of proteins, DNA fragments, or viruses^11,12^.

Plasmonic gratings have also been extensively studied for other purposes such as controlling the emission of electromagnetic (infrared and terahertz) waves, fluorescence enhancement, and generation of surface-enhanced Raman scattering (SERS) effects^5,13–16^. Various methods have been developed to fabricate plasmonic gratings using nanolithography techniques, such as laser interference lithography, thermal nanoimprinting, and dry or wet etching^13,15,17^. However, the resonance frequency or wavelength of the plasmon in plasmonic grating, and hence its SPR spectrum, cannot be easily estimated. For a conventional flat metal layer coupler, such as that in the Kretschmann configuration, the SPR spectrum can be calculated with high accuracy simply by using the Fresnel formula in conjunction with the transfer matrix method^18,19^.

Despite the advantages mentioned above, the development of grating-coupled SPR systems still faces two challenging problems: (1) the design and fabrication of the plasmonic grating structure and (2) the theoretical-computational modeling and analysis to predict the SPR spectrum. In the early phase of the study, SPR gratings were mostly fabricated from metal gratings on a flat dielectric layer or vice versa, which requires a thickness and periodicity of only a few tens of nanometers to prevent strong light absorption by the metal structure and meet the resonance condition to achieve optimal SPR excitation^13,20,21^. For this type of all-metallic SPR grating structure, considerable effort has been made to predict the SPR wavelength and understand the plasmonic excitation modes formed on the structure. The SPR wavelengths are reported primarily in the infrared region (2.5–10 µm), which is useful for infrared spectroscopy applications. However, when the application in the visible light region becomes a concern, for all-metallic SPR gratings, the periodicity should be reduced to ~ 100 nm, whereas the groove width is only on the order of a few to tens of nanometers. Such structures are still not easy to be fabricated or manufactured with the current established technology and thus many reports are still based on computational work^22–24^. Nevertheless, these studies have provided a fundamental understanding of the plasmonic excitation mode in terms of the periodicity, distance between the nanolines or ridges, width and depth of the groove or trench, and other geometric parameters in all metallic grating structures. In addition to the propagating plasmon mode, it has also been predicted the plasmon excitation inside the groove, which is generated when the groove width is only a few tens of nanometers but the groove depth must be many times greater than its width^22–24^. Although it might be directly applicable in some applications, such as controlling infrared emissions, it is unlikely that it can be used for practical sensing applications by filling or immobilizing molecules or analytes inside the groove structure^25,26^.

For a metal-coated dielectric layer with surface relief or corrugated structure, the periodicity and corrugated structure dimensions of the grating can be extended to several hundred nanometers^27,28^. In addition to utilizing groove sections with the filling or immobilization of sensing or analyte molecules, the structure can also be fabricated using various methods mentioned above^5,28,29^. Nanoimprint lithography (NIL) offers several advantages over other techniques for mass production, and it is widely used for the fabrication of Bragg gratings in fiber-optic communications, fiber-optic sensors, and micro/nano fluidics. For such all-dielectric grating structures, the Bragg diffraction wavelength can be accurately predicted by both theoretical and computational studies. The simplest approach is by introducing an effective refractive index into the surface relief or corrugated structures^30^. In contrast, due to the presence of a metallic layer, the resonance wavelength or SPR spectrum of a plasmonic grating cannot be straightforwardly predicted. Therefore, several studies have been conducted for this purpose by performing modeling and simulation calculations using the finite element method (FEM), finite difference time domain (FDTD), and rigorous coupled wave analysis (RCWA) methods^31–35^. In this paper, we report on the fabrication of one-dimensional (1D) plasmonic gratings with rectangular wave-shaped surface relief (RSR-PG) using a simple nanoimprint lithography technique and the analysis of their SPR characteristic spectra through both experimental characterizations and simulation computations. The SPR spectral characteristics indicate the presence of dual-mode surface plasmons, that is, a long-range SPR, which is formed along the grating surface, and a quasi-LSPR, which is formed within the groove section. Moreover, when the groove width was quite narrow, the plasmonic surface lattice resonance (SLR) was also identified.

## Methods

### Fabrications of RSR-PG structures

**Figure 1:**
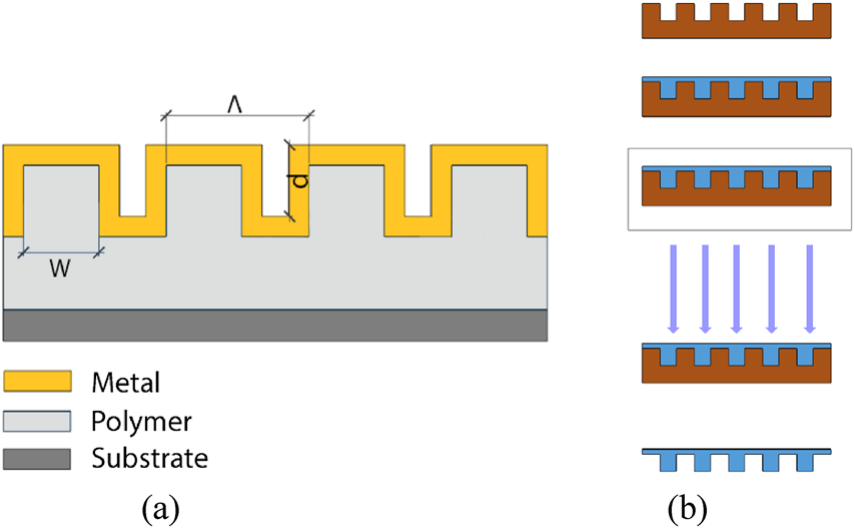
(**a**) Schematic of RSR-PG structure fabricated in this study. (**b**) Nanoimprinted photocuring lithography employed in this study.

A typical RSR-PG structure fabricated in this study is shown in Fig. 1a. The structure formed a 1D dielectric (polymer) surface-relief grating covered by a gold (Au) nanolayer. The polymer precursor used in the present work was prepared from 3-trimethoxysilyl propyl methacrylate (TMSPMA) via the sol–gel route, as reported elsewhere^36^. Figure 1b schematically shows the typical preparation process of these polymer grating structures using nanoimprint photocuring lithography. A silicone template with a grating structure having a periodicity of 700 nm and depth of 350 nm was used in this study. The precursor gel was then dropped onto the silicone template and placed in a vacuum chamber for 15 min. The chamber was then illuminated with a UV lamp for 15 min. The resulting replica structure was separated from the template and attached to a glass slide, which was then finally metalized with gold (Au) by the thermal evaporation method.

### Spectra measurements

**Figure 2:**
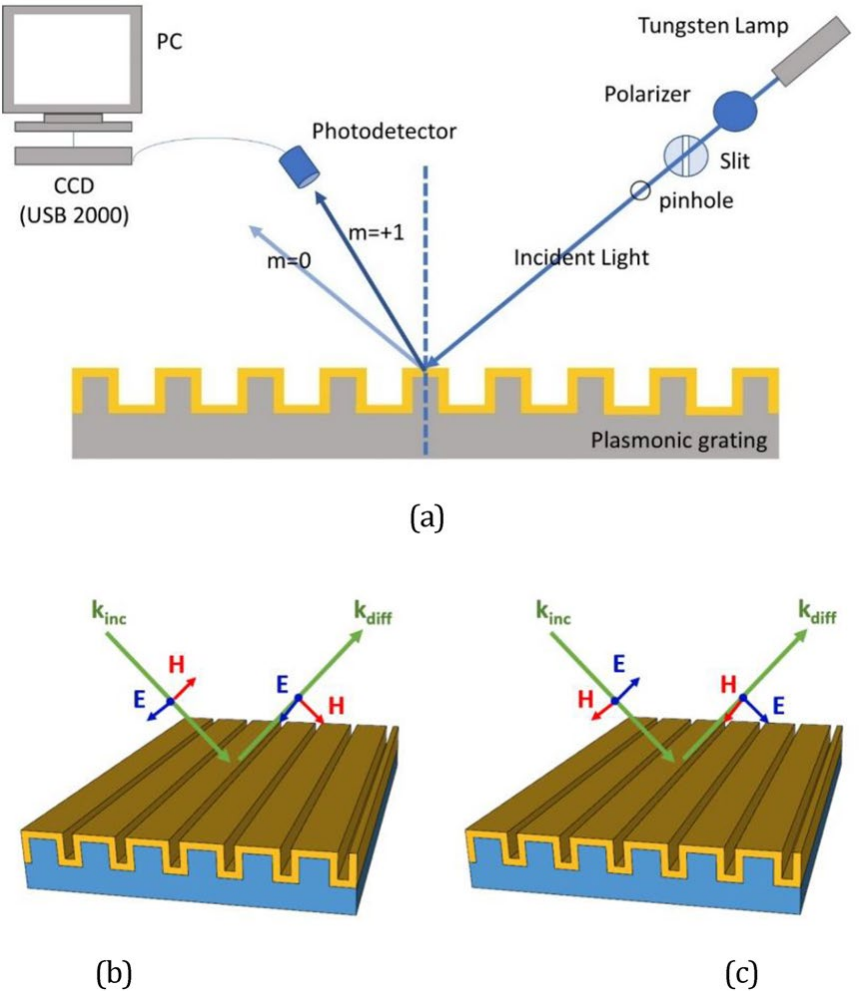
(**a**) Schematic diagram of the SPR spectral measurement setup. Illustration of E and H field polarization in (**b**) TE mode (**c**) TM modes.

Reflectance spectra were measured using a CCD spectrophotometer (USB 2000, Ocean Optics) and a white light source (halogen tungsten lamp) polarized in two different polarizations, namely transverse electric (TE) and transverse magnetic (TM) polarization. TE polarization is a condition in which the electric field polarization of the incident light is parallel to the grating lines, or in other words, perpendicular to the incident plane. In TM polarization, the electric field polarization is parallel to the incident plane. Figure 2a shows a diagram of the SPR spectral measurement setup.

### Simulation

The reflection spectra and field distributions were simulated using the RCWA method, a semi-analytic computational method that implements the Fourier transform in its formulation^31–35^. This method is then very suitable for calculating light propagation in periodic structures, such as the plasmonic grating investigated here. Here, we performed a simulation for two different incident beams, TE and TM polarizations, as illustrated in Fig. 2b,c. For a simple flat metal/dielectric structure, an SPR dip is observed only in the TM mode, in which a plasmon wave is formed along the surface with an electric field perpendicular to the surface (in the z-axis direction)^18^.

**Figure 3:**
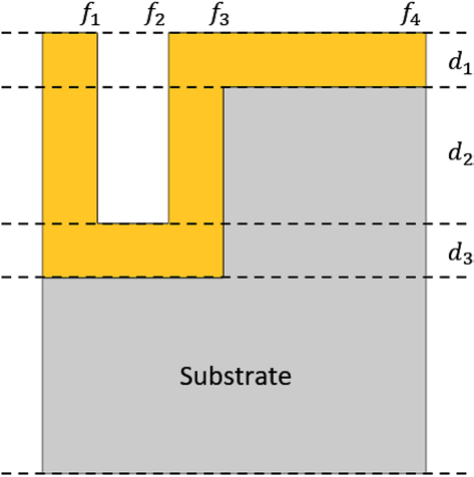
A 1D periodic surface relief structure (with a thin layer of Au on the top) that is used in the computation of the SPR spectra simulation. This structure imitates the fabricated RSR-PG structures.

The RCWA method calculates light-wave propagations by transforming Maxwell’s equation into a matrix formulation using permittivity and electric/magnetic fields in their Fourier series representations^34,35^. For a planar periodic structure in the *x–y* plane, the dielectric permittivity and magnetic permittivity of the medium can be expressed as a Fourier series expansion as follows:

1 $$\varepsilon_{r} \left( {x,y} \right) = \sum\limits_{m = - \infty }^{\infty } {\sum\limits_{n = - \infty }^{\infty } {a_{m,n} e^{{ - j\left( {\frac{2\pi mx}{{\Lambda_{x} }} + \frac{2\pi my}{{\Lambda_{y} }}} \right)}} } } \;{\text{and}}\;\mu_{r} \left( {x,y} \right) = \sum\limits_{m = - \infty }^{\infty } {\sum\limits_{n = - \infty }^{\infty } {b_{m,n} } } e^{{j\left( {\frac{2\pi mx}{{\Lambda_{x} }} + \frac{2\pi ny}{{\Lambda_{y} }}} \right)}} ,$$

where *a*_*m,n*_ and *b*_*m,n*_ are Fourier expansion coefficients. The solution of the electric and magnetic fields can then also be written as a Fourier series given by

$${E}_{x}\left(x,y,z\right)={\sum }_{m=-\infty }^{\infty }{\sum }_{n=-\infty }^{\infty }{s}_{x}^{m,n}\left(z\right) {e}^{-j\left[{k}_{z}\left(m\right)x+{k}_{y}\left(n\right)y\right]},$$

$${E}_{y}\left(x,y,z\right)={\sum }_{m=-\infty }^{\infty }{\sum }_{n=-\infty }^{\infty }{s}_{y}^{m,n}\left(z\right) {e}^{-j\left[{k}_{z}\left(m\right)x+{k}_{y}\left(n\right)y\right]},$$

2 $${E}_{z}\left(x,y,z\right)={\sum }_{m=-\infty }^{\infty }{\sum }_{n=-\infty }^{\infty }{s}_{z}^{m,n}\left(z\right) {e}^{-j\left[{k}_{z}\left(m\right)x+{k}_{y}\left(n\right)y\right]},$$

where *s* is the Fourier series coefficient of the electric field *E* in each axis direction. The magnetic field *H* can also be expressed in a similar expansion, where u represents the Fourier series coefficients. In short, by substituting the Fourier series expansions into ﻿$\nabla \times {\varvec{E}}=-\frac{\partial {\varvec{B}}}{\partial t}$ and $\nabla \times {\varvec{B}}={\mu }_{0}{\varepsilon }_{0}\frac{\partial {\varvec{E}}}{\partial t}$, the following one-dimensional wave propagation for the TM mode can be obtained as follows

$$\frac{{d}^{2}}{dz}{u}_{y}-{\Omega }^{2}{u}_{y}=0,$$

3 $${\Omega }^{2}=PQ,$$

where the detail descriptions for Ω, P, and Q can be seen in the Supplementary Information. This equation is a matrix wave equation that can be solved solely by computation. A similar form can also be obtained for the TE mode. The program code for these computations was adopted from the RCWA code in MATLAB written by the Zhang group of the Georgia Institute of Technology^34,35^. The program code was modified to adopt a periodic structure that imitates the fabricated structures consisting of five layers, as shown in Fig. 3. The relative electric permittivity of each layer was expressed as a Fourier series, in which the series coefficients for the *n*-th expansion order are given by

4 $${a}_{m}=\frac{i}{2\pi m}\left[{\varepsilon }_{{f}_{1}}\left({e}^{-\frac{i2\pi m}{\Lambda }{f}_{1}}-{1}\right)+{\varepsilon }_{{f}_{2}}\left({e}^{-\frac{i2\pi m}{\Lambda }{f}_{2}}-{e}^{-\frac{i2\pi m}{\Lambda }{f}_{1}}\right)+{\varepsilon }_{{f}_{3}}\left({e}^{-\frac{i2\pi m}{\Lambda }{f}_{3}}-{e}^{-\frac{i2\pi m}{\Lambda }{f}_{2}}\right)+{\varepsilon }_{{f}_{4}}\left({e}^{-\frac{i2\pi m}{\Lambda }{f}_{4}}-{e}^{-\frac{i2\pi m}{\Lambda }{f}_{3}}\right)\right],$$

$${a}_{0}=\frac{1}{\Lambda }\left[{\varepsilon }_{{f}_{1}}({f}_{1})+{\varepsilon }_{{f}_{2}}({f}_{2}-{f}_{1})+{\varepsilon }_{{f}_{3}}({f}_{3}-{f}_{2})+{\varepsilon }_{{f}_{4}}({f}_{4}-{f}_{3})\right],$$

where ﻿${f}_{i}$ (*i* = 1,2,3,4) are the coordinates of the end position of the *i*-th segment in each layer.

## Results and discussions

### Long-range surface plasmon resonance characteristics

**Figure 4:**
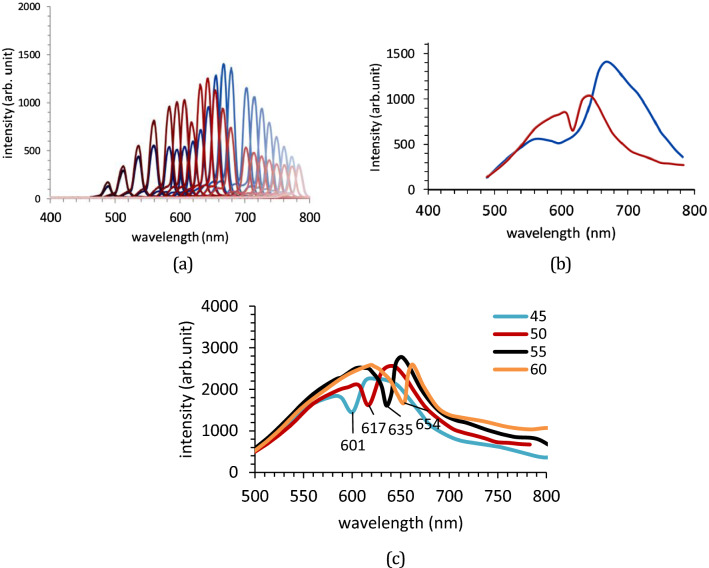
(**a**) Reflectance spectra of the diffracted beams measured for TE (blue line) and TM (red line) polarizations at an incident angle of 50°. (**b**) SPR spectra obtained from the spectral envelope of the spectra shown in Figure (**a**). (**c**) The reflectance spectra show spectral dip shifting with increasing incident angle from 45° to 50°, 55°, and 60°.

Figure 4a shows the reflectance spectra measured from the diffracted beams of an RSR-PG sample. The RSR-PG structure was placed at various incident angles, namely 45°, 50°, 55°, or 60° normal to the RSR-PG surface. The photodetector then sequentially measured the reflectance beam at various angles normal to the RSR-PG surface, corresponding to the second-order diffraction angles. At each photodetector angle, a narrow reflection band spectrum was seen, which is a typical characteristic of a diffracted beam. All these individual diffraction spectra were plotted together in the same figure, producing a typical reflectance spectrum, as shown in Fig. 4a. We can easily notice a spectral envelope shape in both the TE and TM polarization spectra, as shown in Fig. 4b. A remarkable spectral dip is observed in the TM spectrum, but not in the TE spectrum, which can be assigned as a dip originating from the SPR wave excitation inside the RSR-PG structure coupled to a diffraction mode. The dip shifts from approximately 600 nm to 655 nm as the incident angle increases from 45° to 60°, corresponding to a frequency shift from 3.14 × 10^15^ Hz to 2.88 × 10^15^ Hz. The popular light diffraction formula given by

5 $${n}_{dm}\mathrm{sin}{\theta }_{dm}={n}_{inc}\mathrm{sin}{\theta }_{inc}- m\frac{\uplambda }{\Lambda },$$

where *n*_*dm*_ is the refractive index medium in which the diffracted light travels, *n*_*inc*_ is the refractive medium in which the incident light travels, *θ*_*dm*_ is the diffraction angle of the *m*-th order, *θ*_*inc*_ is the incident angle, λ is the wavelength of light, and Λ is the grating periodicity. Here, both the incident and reflected beams travel in the same medium above the grating surface, that is, simply air atmosphere; then, *n*_*dm*_ = *n*_*inc*_ = 1. Based on this equation, the spectrum dips can be attributed to the diffraction order m =  + 1, as illustrated in Fig. 2a.

In general, an SPR wave in a metallic grating structure, the resonance occurs between an incident electromagnetic wave and electrons oscillation on the metal/dielectric interface, where the resonance condition is coupled to the *m*-th diffraction order (${k}_{xm}$), given by^37,38^

6 $${k}_{xm}=\frac{2\pi {n}_{inc}\mathrm{sin}{\theta }_{inc}}{{\lambda }_{inc}}- m\frac{2\pi }{\Lambda }={k}_{sp},$$

where ${k}_{xm}$ represents the light propagation constant along the grating, whereas ${k}_{sp}$ is the propagation constant of the plasmon wave given by

7 $${k}_{sp}=\frac{\omega }{c}\sqrt{\frac{{\varepsilon }_{d}{\varepsilon }_{m}}{{\varepsilon }_{d}+{\varepsilon }_{m}}},$$

where ${\varepsilon }_{m}$ is the metal permittivity and ${\varepsilon }_{d}$ is the dielectric permittivity of the superstrate. The resonance wavelength (at a particular λ_*inc*_) increases with an increase in the incident angle.

**Figure 5:**
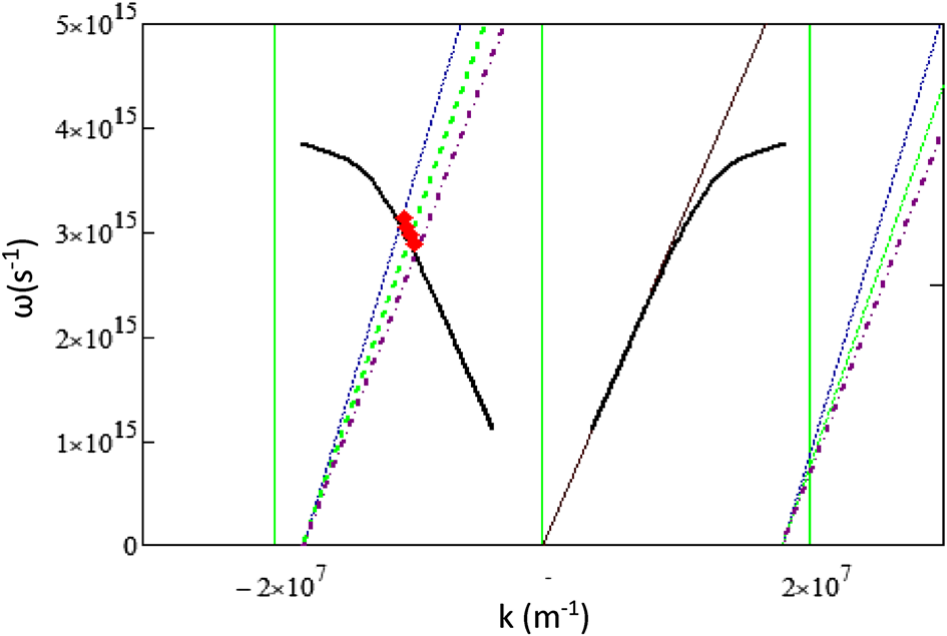
The curve intersection representing surface plasmon resonance condition as the intersection of the two curves given by the diffraction formula in Eq. (6) and the dispersion relation in Eq. (7) for diffraction orders m =  − 2 and m =  + 2. The experimental data are shown by “red filled diamond” symbol for comparison.

Figure 5 shows the intersection points of the two curves with the resonance conditions given by Eqs. (6) and (7). The resonance conditions obtained from the above experimental data are indicated by the red diamonds. The experimental data then perfectly matches the plasmon wave excitation that is coupled with the diffraction order *m* =  − 2. Therefore, interestingly, while the measured spectral dip is related to the diffraction order m =  + 1, the SPR wave formed on the grating is related to the diffraction order m =  − 2. However, this condition can be understood if we consider that, in a periodic structure, the light beam will be decomposed into a number of orders (*m*) of its fundamental harmonic wave, as given by Eq. (2). In this case, it is the second-order (m =  − 2) diffraction that actually being in resonance with the grating plasmon *k*_*sp*_. The incident light (electromagnetic) energy is then strongly absorbed, and as a consequence of energy conservation, significantly suppresses the light intensity of the diffracted beam of *m* =  + 1. Therefore, the plasmon resonance is not due to direct coupling with the diffraction order *m* =  + 1, but with *m* =  − 2.

**Figure 6:**
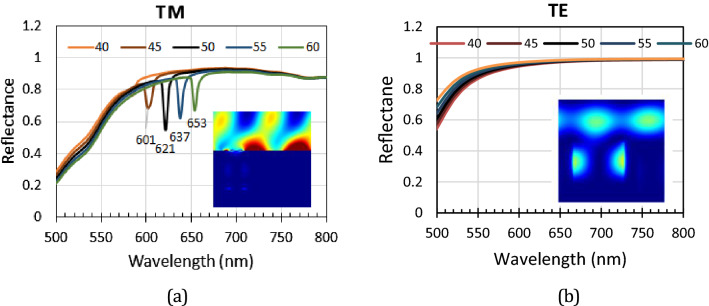
The calculated reflectance spectra of the plasmonic surface relief grating, with the periodic structure unit shown in Fig. 3 for the (**a**) TM mode and (**b**) TE mode at various incident angles. Inset: figures showing the E_z_ field intensities calculated for a 50° incident angle.

While Eq. (6) is useful for the rapid evaluation and verification of the experimentally observed resonance wavelength, it cannot accurately predict the resonance wavelength for a complex structure. In addition to the periodicity, the resonance condition is also affected by other factors, including the depth, width, and shape of the groove, dielectric permittivity, and thickness of each structure layer. The distribution of the formed plasmon field is also affected by those parameters, which are important in applications that provide optimum overlap between the plasmon field and the analyte or sensed molecular layer. Therefore, in this study, it was necessary to perform a simulation computation based on the RCWA method to obtain a more accurate result for estimating the resonance wavelength and plasmon wave distribution. Figure 6a shows the reflectance spectra calculated for the TM mode from the structure shown in Fig. 3, with Λ = 700 nm, *a*_1_ = 105 nm, *a*_2_ = 265 nm, *a*_3_ = 370 nm, *a*_4_ = 700 nm, *d*_1_ = 130 nm, *d*_2_ = 285 nm, and *d*_3_ = 65 nm. Here, we assume that the Au thickness on the top of the ridge is larger than that at the bottom of the groove by considering the possibility of different amounts of deposited Au reaching those areas during the evaporation process. The dielectric permittivity of the ridge part was set to 1.5 and was wavelength-independent. The metal was set as gold (Au) with wavelength-dependent complex permittivity, which was taken from the experimental data reported by Palik^39^. The reflectance spectra dips of those simulation results are similar to those of the experimental results shown in Fig. 4c. The dips were observed at 601, 621, 637, and 653 nm for incident angles of 45°, 50°, 55°, and 60°, respectively. In TE mode, as shown in Fig. 6b, neither the experimental nor calculation results show a dip that can be associated with SPR excitation. These facts confirm that the observed dips are due to plasmon polaritons or SPR wave excitation in the structure. Notably, this SPR wave can be formed only in a certain wavelength region (and hence at a certain incident angle range), which cannot be predicted by the simple relationship given by Eq. (6). This result clearly shows the agreement between the RCWA-based computation results and experimental data.

The insets in Fig. 6a,b show the distribution of the Ez field for an incident angle of 50° in TM mode. For the Ez field, the distribution of the intense electric field perpendicular to the Au layer can be associated with surface plasmon formation. Compared to other wavelengths, at the dip (resonance) wavelength, the distribution of the Ez field at the ridge surface is the strongest. Therefore, we can confirm that surface plasmons are formed but appear only at the ridge surface and not inside the groove section. In addition, this situation is obviously different from that found in the TE mode case, where no strong Ez field distribution is observed at the ridge surface, as shown in Fig. 6b.

### Quasi-localized surface plasmon characteristics

**Figure 7:**
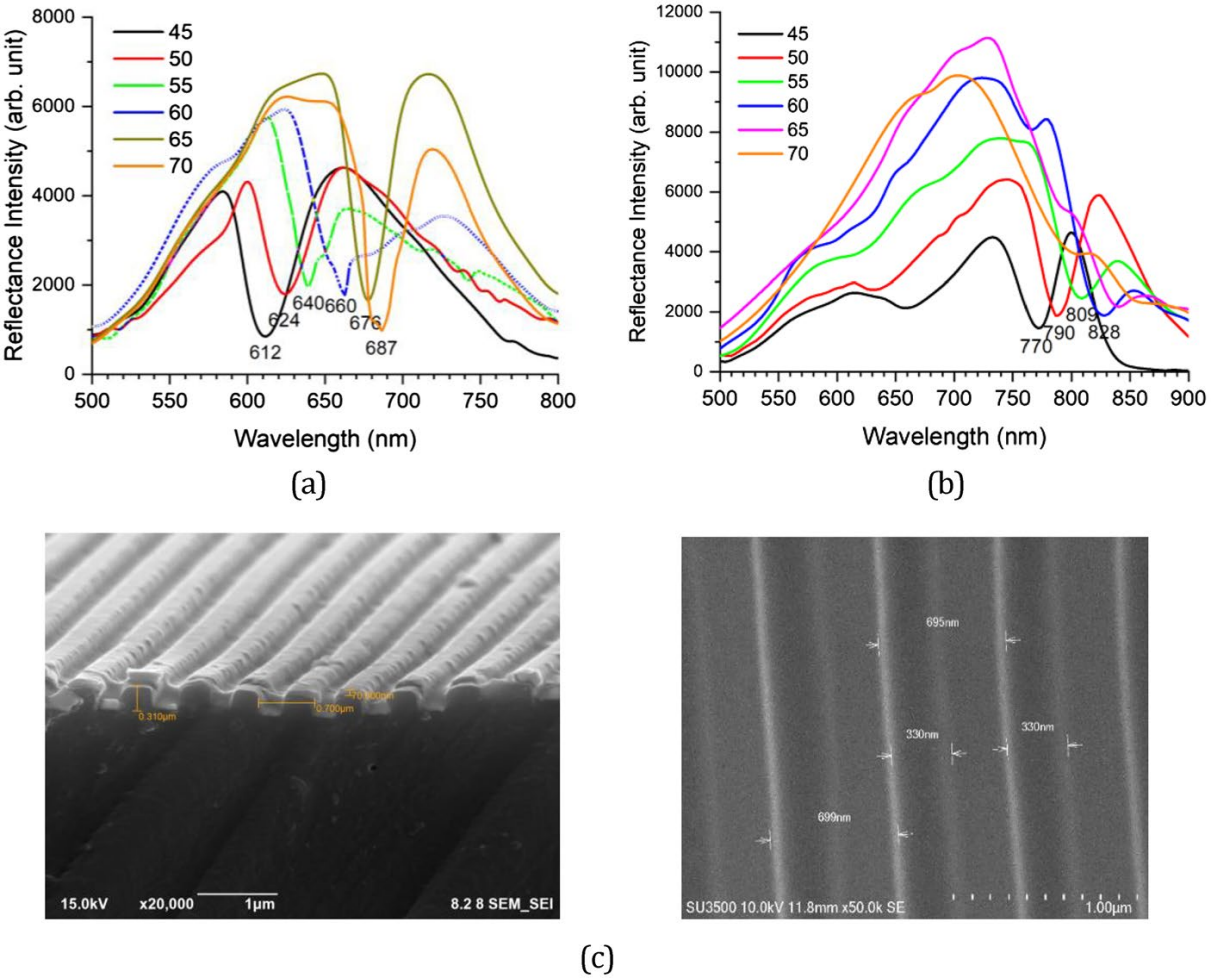
Reflectance (SPR) spectra measured from a RSR-PG sample (**a**) before and (**b**) after being covered with olive oil. (**c**) FE-SEM images measured from this RSR-PG sample. The image surface of the polymer grating before covered by Au nanolayer (displayed to show highly uniform and dense polymer surface).

To confirm the possibility of plasmon wave excitation inside the groove, SPR spectra have been measured for an RSR-PG sample that was previously spin-coated with olive oil to form a thin layer of organic liquid on its surface. Figure 7a,b show the experimental data of the reflectance spectra obtained from an RSR-PG with and without spin-coated olive oil, respectively. Both spectra show SPR dips, but appear at different wavelength ranges. In comparison to the RSR-PG before being covered with olive oil, after being covered with olive oil, the SPR dips are shifted to a longer wavelength, that is, in the range of 770 nm to 830 nm for the incident angle changing from 45° to 60°. Figure 7c shows the FE-SEM image of this RSR-PG sample, indicating a ridge height of 310 nm and an Au layer thickness of 70 nm. In order to confirm the presence of olive oil on the top of this RSR-PG, after being spin coated with olive oil, Raman scattering spectroscopy measurements were carried out. Figure S1 shows typical characteristics of the Raman spectra of olive oil^40^. To better understand the origin of the observed spectral dip shifts, the experimental data were compared to the SPR spectral simulation.

**Figure 8:**
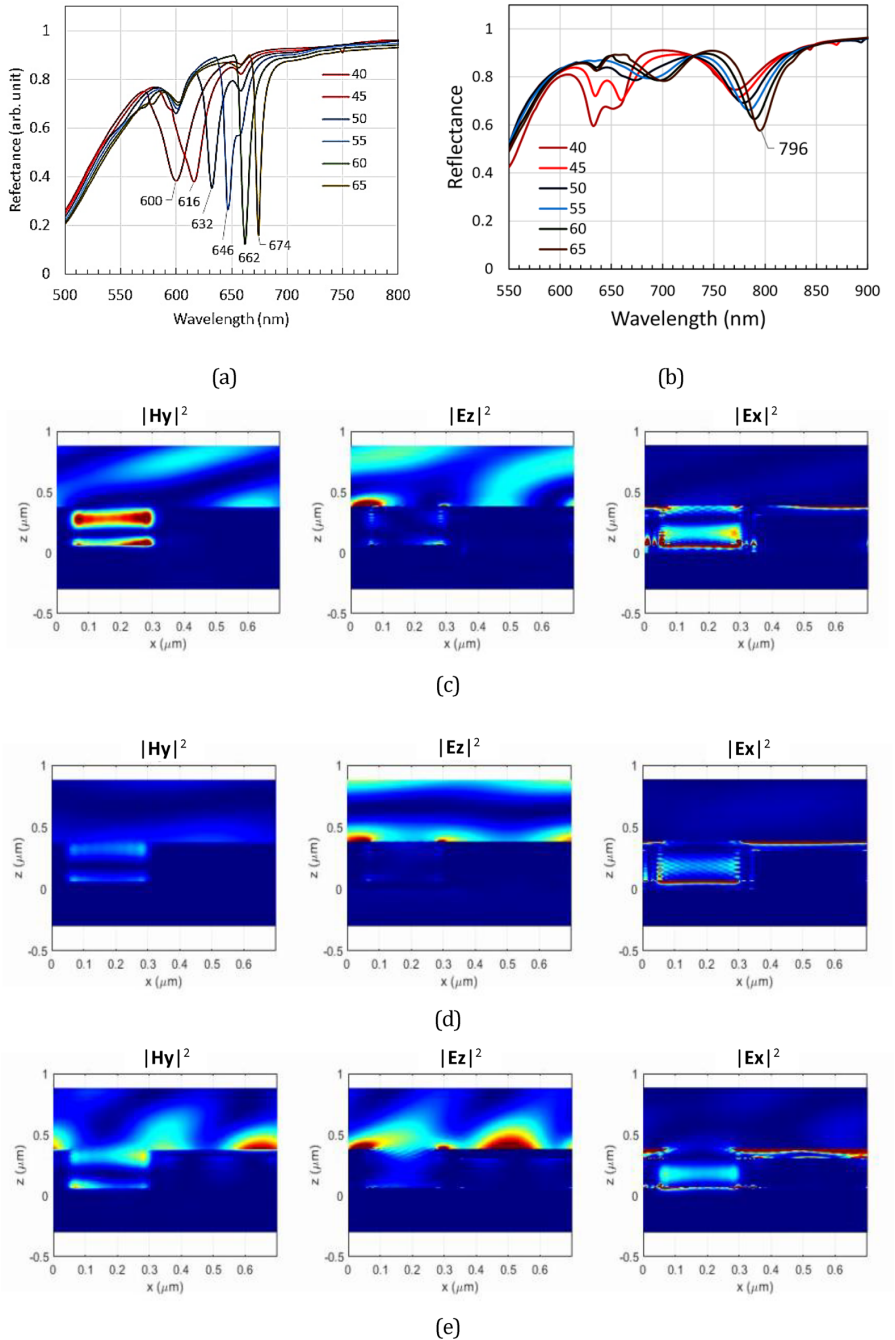
(**a**) SPR spectra simulation calculated for RSR-PG without a thin liquid layer on its surface. (**b**) Similar to figure (**a**) but with a thin liquid layer on its surface and inside the groove section. (**c**) Magnetic and electric field intensities calculated for the spectra (b) at λ_inc_ = 778 nm. (**d**) Similar field intensities calculated for the spectra (**b**) at λ_inc_ = 900 nm. (**e**) Field intensities simulation of spectra (**a**) calculated at λ_inc_ = 632 nm.

Figure S2 shows the simulation of SPR spectral shifts for the case where a liquid forms a dielectric layer covering the RSR-PG surface without filling the groove section. The refractive index varies from 1.0 to 1.6. The SPR dips shift to the range 680–730 nm in the presence of a thin layer of liquid with a refractive index of 1.5. However, this range is still much lower than that observed in the experimental data shown in Fig. 7b. We then suggest that the observed experimental data might be related to the filling of the groove section with olive oil. The SPR spectra simulation was then calculated using the same method as described above with the same periodicity of 700 nm but different parameters related to the groove depth and Au layer thickness (namely *a*_1_ = 60 nm, *a*_2_ = 290 nm; *a*_3_ = 350 nm, *a*_4_ = 700 nm, *d*_1_ = 75 nm, *d*_2_ = 242.5 nm, and *d*_3_ = 67.5 nm). Figure 8 shows the simulation results. For the RSR-PG without a particular dielectric layer on top of it, which means that it is simply in contact with air (*n* = 1), the dips appear in the range of 616–662 nm for the incident angle from 45° to 60°. For an RSR-PG in which a dielectric layer with refractive index *n* = 1.5 covered its surface and filled its groove, the SPR dips appeared in the range of 772–796 nm for the incident angle from 45° to 60°. The simulation for the case with the filled groove section then produced a result much closer to the experimental data than the empty groove section case. Therefore, both the experimental data and simulation results confirmed SPR generation inside the groove rather than on the RSR-PG surface.

To further confirm that the SPR dip at ~ 800 nm arose from SPR excitation inside the groove, calculations of the electric-field intensity distribution were also performed for this structure. As noticeably seen in Fig. 8c, a strong magnetic field **H**_**y**_ intensity appears inside the groove at an incident light wavelength of 778 nm and an incident angle of 50 nm. The wavelength and incident angle corresponded to the SPR dip in the spectrum shown in Fig. 8b. The magnetic field **H**_**y**_ distribution is important because it is related to the two electric field components **E**_**x**_ and **E**_**z**_, where **E**_**x**_ is perpendicular to the surface of the groove wall. Therefore, inside the groove, the appearance of strong **H**_**y**_ and **E**_**x**_ intensities can indicate an SPR field excitation. Figure 8c shows the appearance of a strong **E**_**x**_ field intensity distribution inside the groove. In contrast, beyond the SPR regime, such as at a wavelength of 900 nm, strong **H**_**y**_ and **E**_**x**_ intensities are not observed inside the groove, as shown in Fig. 8d. Similarly, for the case where SPR occurs only at the RSR-PG surface, as shown in Fig. 8e, strong **H**_**y**_ and **E**_**x**_ intensities are not observed inside the groove section, although a strong **E**_**z**_ intensity distribution is observed on the RSR-PG structure surface. This field intensity distribution appears for incident light at a wavelength of 632 nm and incident angle of 50º, which corresponds to the SPR dip in Fig. 8a.

It may also be interesting to address two additional important features before drawing conclusions. The first is related to the width of the SPR dip. The SPR spectra shown in Fig. 4c exhibit a narrower dip width than the spectra in Fig. 7a, that is, 11 nm and 30 nm, respectively. The simulation results for the spectra in Fig. 4c are shown in Fig. 6a, where the spectra were calculated for an RSR-PG structure with a 130 nm Au layer, corresponding to a narrow groove width of only 105 nm. On the other hand, for the spectra in Fig. 7a, the simulation spectra in Fig. 8a were obtained from an RSR-PG structure with a 65 nm Au layer, which indicates a wider groove width of 230 nm. It is plausible that the plasmon excitation in the first case, which occurred in the RSR-PG structure with a narrow groove width, was also enhanced by the plasmonic surface lattice resonance (Plasmonic SLR) phenomenon. Plasmonic SLR is a unique phenomenon observed in an array of nanoparticles that results in a much narrower SPR dip than the individual LSPR dip^41–43^. This phenomenon can also occur in the present case, as indicated by the experimental and simulation results. The width of the Plasmonic SLR dip is determined by the quality factor of resonance Q, which is intrinsically linked to the ratio of stored energy to energy lost in this plasmonic resonance, given by Q = λ_min_/Δλ (where λ_min_ is the resonance wavelength and Δλ is the SPR dip width)^41^. It is apparent that the first RSR-PG with a narrow SPR dip has a higher Q factor than the other owing to the smaller distance between the ridges and a larger groove depth.

**Figure 9:**
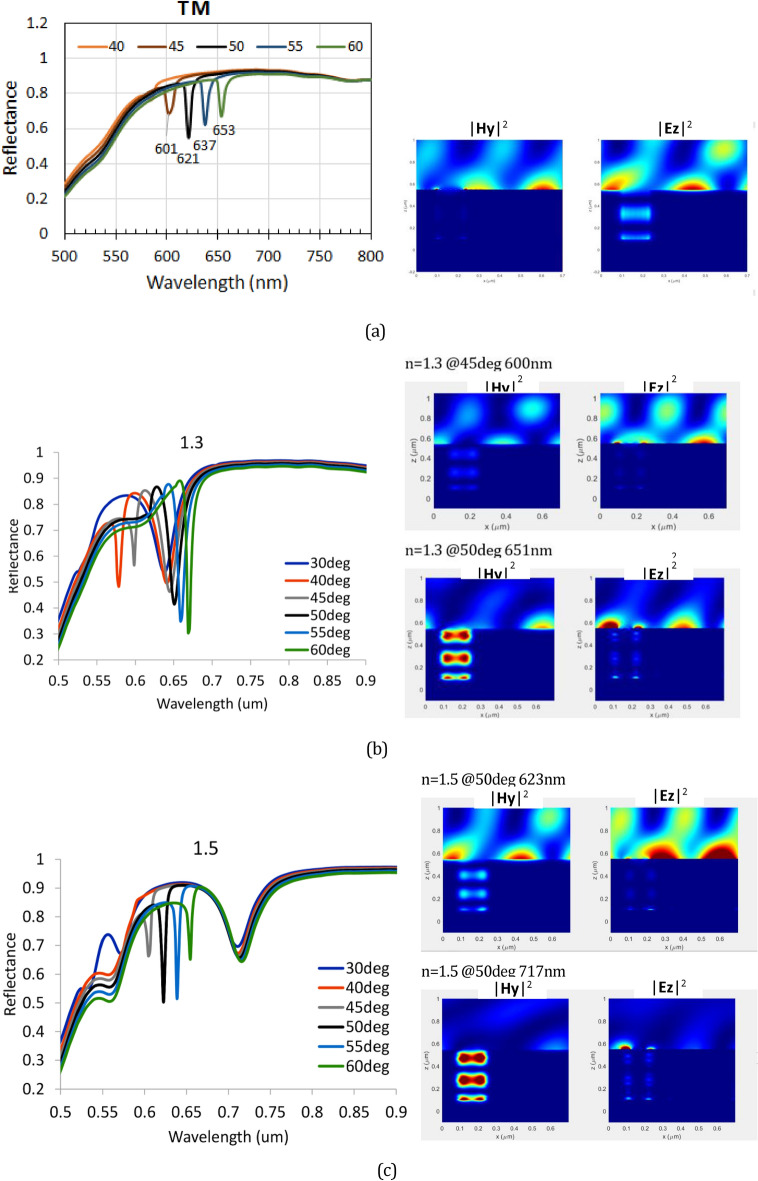

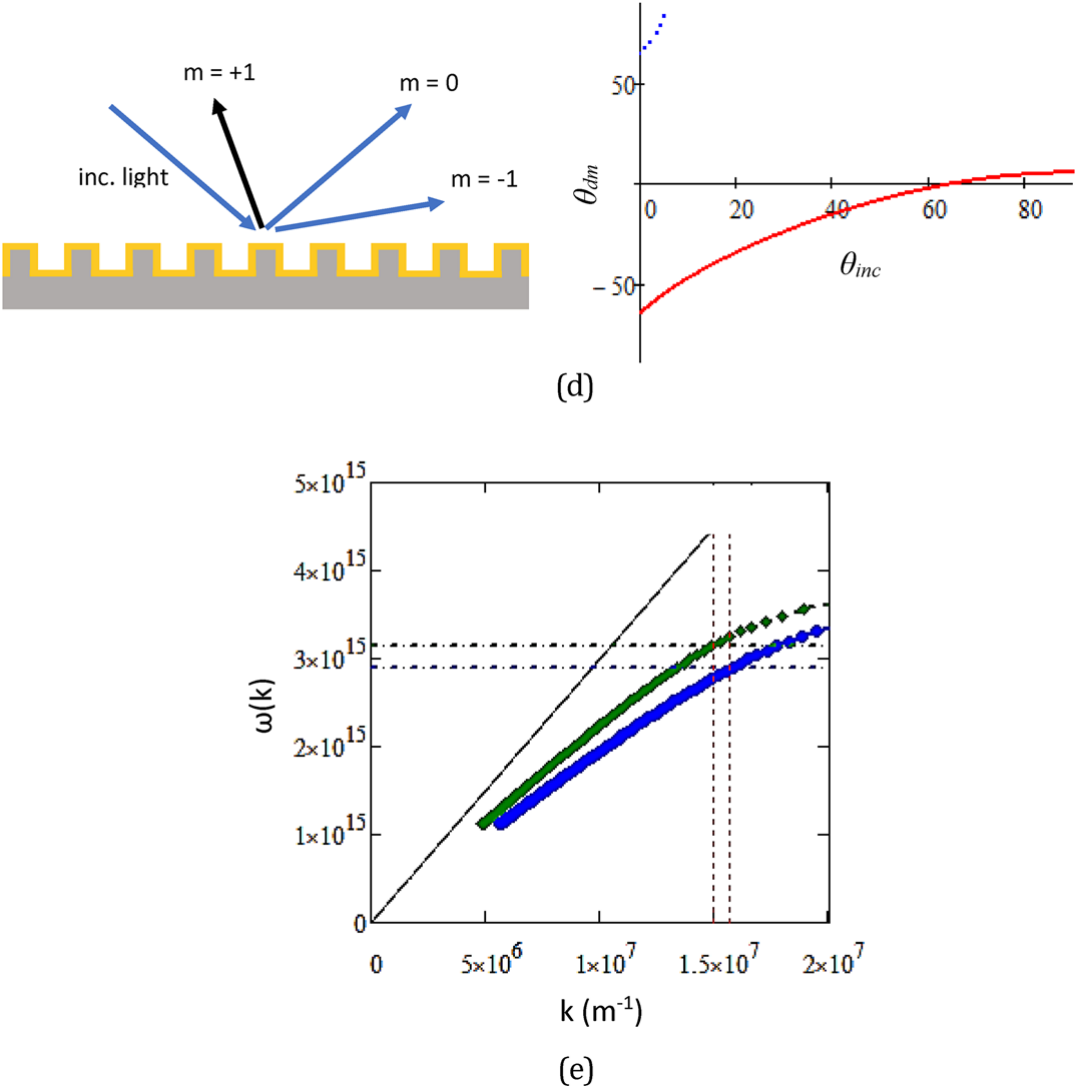
The reflectance spectra of the plasmonic surface relief grating with a groove section filled with a medium with a refractive index of (**a**) n_groove_ = 1.0, (**b**) n_groove_ = 1.3, where the broad dip around 650 nm may be assigned as LSPR, and (**c**) n_groove_ = 1.5, where the broad dip around 715 nm may be assigned as LSPR. The Hy and Ez field intensities are also shown that were calculated at an incident angle of 50° and at the wavelengths indicated in the figure. (**d**) Illustration of diffracted beam m =  + 1 that was measured in the spectra measurements and the calculated diffraction angle for m =  + 1 as a function of incident angle θ_inc_. (**e**) Dispersion relation curve for plasmons at the metal/dielectric interface, representing the groove wall, for n_groove_ = 1.3 (green symbol) and n_groove_ = 1.5 (blue symbol). The horizontal line is the line correlating the plasmon wavelength and free-space light incident frequency.

The second important feature is related to the possibility of LSPR-like excitation inside the groove section. Figure 9 shows the simulation of the SPR spectra obtained from the same structure used to produce Fig. 6a. However, the groove section is supposed to be filled with a liquid with a refractive index of *n*_*groove*_ = 1.3 and 1.5. For clarity, the SPR spectra for *n*_*groove*_ = 1 (i.e., for the case with no liquid on the grating surface and inside its groove) are also shown in the figure. There are two different types of dips: a narrow dip, which shifts significantly with increasing incident light angle, and a wider dip, which is almost independent of the incident light angle. With an increase in *n*_*groove*_, both the narrow dip and the wider dip become red-shifted. For the empty groove (*n*_*groove*_ = 1), the wider dip was not observed because this dip may appear in the blue or UV light range, but the light absorption of Au is very strong in that range.

For *n*_*groove*_ = 1.5 and *λ*_o_ = 623 nm, which corresponds to the narrow SPR dip, the **E**_**z**_ field intensity is strong at the ridge surface, but the **H**_**y**_ field intensity inside the groove section is weak. Oppositely, for *λ*_o_ = 717 nm, the **E**_**z**_ field intensity was weak at the ridge surface, but the **Hy** field intensity was strong inside the groove. This indicates that the narrow dip may be associated with the long-range plasmon wave excitation (long-range SPR) along the grating surface. On the other hand, the wider dip can be attributed to the quasi-LSPR formed inside the groove section, as explained below. First, we consider that this groove section, depending on its geometrical structure and materials, may support LSPR at the gold/dielectric interface of the groove wall. We can then also consider the z-component of the diffracted beam (*k*_*dz*_), at certain incident angle, may be sufficient to match the propagation constant (plasmon momentum) *k*_*sp*_ of the groove wall. When this condition is fulfilled, plasmon resonance occurs, and the incident light energy will be strongly absorbed, leading to the appearance of the SPR dip. Using Eq. (5), the diffraction angle can be calculated and plotted as shown in Fig. 9d. For a simple evaluation, we can see that at an incident angle of approximately 60º, the diffracted beam will be perpendicular to the grating surface and may excite the plasmon field inside the groove. This plasmon can not propagate, however, because it is confined inside the groove structure. The dispersion relation for plasmons on the groove wall surface can be calculated using Eq. (7) and plotted as shown in Fig. 9e. From Fig. 9b,c, the periodicity of Hy intensity for *n*_*groove*_ = 1.5 and groove = 1.3 are estimated to be about 400 nm and 420 nm, which corresponding to *k*_*sp*_ = 1.57 × 10^7^ m^−1^ and *k*_*sp*_ = 1.50 × 10^7^ m^−1^, respectively. From Fig. 9e, those values correspond to the incident light frequency of 2.77 × 10^15^ s^−1^ and 3.14 × 10^15^ s^−1^, or equivalent with wavelength of 680 nm and 600 nm, for *n*_*groove*_ = 1.5 and groove = 1.3, respectively. These resonance wavelengths are close to the LSPR dips shown in Fig. 9b,c, which can verify the formation of LSPR inside the groove. There is a discrepancy between those estimated resonance wavelengths and the experimental data; however, this is reasonable because the dispersion relation curve in Fig. 9e was obtained from a simple formula Eq. (7). For a more accurate prediction, the plasmon frequency of the groove structure can also be formulated and calculated using an approach similar to that applied for a trench structure or cavity gold nanorod (CGNR) structure^22,44^. In CGNR, a plasmon is formed at the core dielectric-metal interface of the nanorod. This type of structure, however, is still only studied through computational works, where the reports indicate similar characteristics to those found in the normal gold nanorod structure^45,46^. That is, the resonance wavelength depends on both the structure size and the aspect ratio (length/diameter). The resonance wavelength is redshifted with increasing aspect ratio. For gold nanorods, the LSPR wavelength can reach around 700–900 nm^47^.

The appearance of both long-range SPR and LSPR is crucially dependent on the geometric structure and the dielectric permittivity of the grating. Both long-range SPR and LSPR shift to longer wavelengths with increasing *n*_*groove*_. The LSPR dip shift is larger, but its range is limited. While the LSPR plasmon cannot be observed for *n*_*groove*_ = 1, for *n*_*groove*_ = 1.3, the LSPR can be observed at a light wavelength *λ*_o_ = 650 nm. This is indicated by the appearance of a wide dip in the SPR spectra and the appearance of the Hy field intensity inside the groove (for *θ*_*inc*_ = 50°), as shown in Fig. 9b. However, for the narrow dip at *λ*_o_ = 600 nm, no strong Hy field intensity was observed inside the groove. Instead, a strong **E**_**z**_ field intensity was observed, indicating a long-range SPR. Moreover, as seen in Fig. 9b, it is apparent that there is a transformation from LSPR to long-range SPR with a change in incident angle. At the present stage, we consider that the transformation is caused simply by the matching condition, as described above. Although there are many reports related to the coupling of closely separated localized plasmon fields, resulting in hybridization and splitting into new plasmonic states, we still cannot determine such possibilities for this system^48,49^. Further detailed experimental and computational studies are required to verify this possibility. For this purpose, it is necessary to use a dielectric material with a refractive index of 1.3 because, as shown in Fig. 9b, this configuration exhibits an overlap between long-range SPR and LSPR.

From a practical point of view, however, such a dual-plasmon excitation mode can be useful in certain applications. As an example of a potential application, it can be implemented in devices to classify different liquids for use in nanofluidic chips. Nanofluidic chips have recently attracted increasing interest for both practical and scientific reasons^50–55^. The liquid must be able to fill micro-nano-meter-sized channels or nano-architectural structures that depend on certain properties of the liquid, such as viscosity, wetting, adhesion-cohesion, and surface tension. Such structures can be building blocks of a nanofluidic chip for monitoring or detecting small-molecule products of a process taking place inside a nanofluidic chip. It should be noted that to perform further studies for practical applications using this kind of RSR-PG, its fabrication method still requires many improvements in terms of fabrication reproducibility, which depends on the quality of the synthesized hybrid polymer used here. Based on our experience, commercially available epoxy or acrylate resins cannot be used for this purpose because they cannot form sufficiently dense nanostructures to prevent the penetration of the deposited Au particles. In such a case, a dense nanostructure forming an abrupt electric permittivity change at the dielectric/metal interface cannot be achieved, which hinders SPR wave excitation at the interface.

## Conclusions

In this study, we investigated the characteristics of plasmon resonance in a surface relief grating-based SPR (RSR-PG) with a square-wave structure through experimental and computational studies, which showed quite different characteristics compared to those observed in a PG with a sine-wave structure. The experimental results were confirmed by the computational work results based on the RCWA method. The RSR-PG structures studied here can provide two modes of plasmonic wave excitations: a long-range SPR wave on the grating surface and a quasi-localized SPR formed inside the groove that is almost independent of the incident angle. In addition, when the groove width is sufficiently small, we can also identify a plasmonic lattice surface resonance phenomenon, as indicated by the appearance of a narrow resonance dip, which is the result of the coupling of long-range SPR and the structural lattice. These plasmons become red-shifted within the visible-light region with an increase in the refractive index of the dielectric medium on the grating surface and inside the groove, which may be useful for applications such as biochemical molecular detection. This type of RSR-PG substrate, which can exhibit dual modes of long-range and quasi-localized SPR excitations, may be beneficial for sensors and other applications.

## Supplementary Information

Supplementary Information.

## Data Availability

The datasets generated during and/or analyzed during the current study are available from the corresponding author on reasonable request.
