# Supplementary material for: Dual plasmonic modes in the visible light region in rectangular wave-shaped surface relief plasmonic gratings
Source: Sci Rep. 2023 Mar 31;13:5274. doi: 10.1038/s41598-023-30083-3 (PMC10066186; doi:10.1038/s41598-023-30083-3)
Supplement: Supplementary file 1 — Supplementary Information. [file 41598_2023_30083_MOESM1_ESM.docx]

# Raman spectrum of an RSR-PG after being spin-coated with olive oil


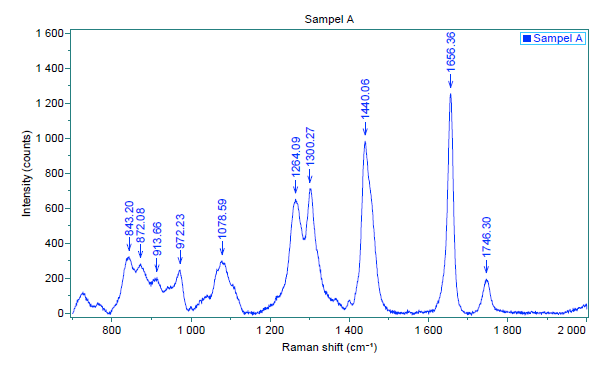


Figure S1. The Raman spectrum measured from an RSR-PG after being spin-coated with olive oil onto its surface.

Figure S1 shows the Raman spectrum measured from an RSR-PG that has been spin-coated with olive oil onto its surface. This Raman spectrum was measured using a LabRAM HR Evolution Confocal Raman Microscope with an excitation laser wavelength of 785 nm. Figure S1 shows typical characteristics of the Raman spectra of olive oil. [^[[1]](#endnote-1)^]

# Simulation of SPR dip shifts

Figure S2. The simulation of SPR dip shifts for the case in which a liquid forms a dielectric layer covering the RSR-PG surface but without filling the groove section.

# Brief description on RCWA method

The RCWA method calculates light-wave propagations by transforming the Maxwell equation into a matrix form and using permittivity and electric/magnetic fields in their Fourier series representations, as briefly described below. [^[[2]](#endnote-2)^,^[[3]](#endnote-3)^] For a planar periodic structure in the *x-y* plane, the dielectric permittivity and magnetic permittivity of the medium can be expressed as a Fourier series expansion as follows:

$\varepsilon_{r}\left( x,y \right)=\sum_{m=-\infty}^{\infty} \sum_{n=-\infty}^{\infty} a_{m,n}e^{-j\left( \frac{2\pi mx}{\Lambda_{x}}+\frac{2\pi mx}{\Lambda_{y}} \right)}$ and

$\mu_{r}\left( x,y \right)=\sum_{m=-\infty}^{\infty} \sum_{n=-\infty}^{\infty} b_{m,n}e^{j\left( \frac{2\pi mx}{\Lambda_{x}}+\frac{2\pi ny}{\Lambda_{y}} \right)}$ eq. 1

where

$a_{m,n}=\frac{1}{\Lambda_{x}\Lambda_{y}}\int_{-\Lambda_{x}/2}^{\Lambda_{x}/2} \int_{-\Lambda_{y}/2}^{\Lambda_{y}/2} \varepsilon_{r}\left(x,y\right)e^{-j\left(\frac{2\pi mx}{\Lambda_{x}}+\frac{2\pi ny}{\Lambda_{y}}\right)}dxdy$ and $a_{m,n}=\frac{1}{\Lambda_{x}\Lambda_{y}}\int_{-\Lambda_{x}/2}^{\Lambda_{x}/2} \int_{-\Lambda_{y}/2}^{\Lambda_{y}/2} \varepsilon_{r}\left(x,y\right)e^{-j\left(\frac{2\pi mx}{\Lambda_{x}}+\frac{2\pi ny}{\Lambda_{y}}\right)}dxdy$

Thus, the solution of electric and magnetic fields can be also written as a Fourier series given by

$$E_{x}\left( x,y,z \right)=\sum_{m=-\infty}^{\infty} \sum_{n=-\infty}^{\infty} s_{x}^{m,n}\left( z \right) e^{-j\left[ k_{z}\left( m \right)x+k_{y}\left( n \right)y \right]}$$

$$E_{y}\left( x,y,z \right)=\sum_{m=-\infty}^{\infty} \sum_{n=-\infty}^{\infty} s_{y}^{m,n}\left( z \right) e^{-j\left[ k_{z}\left( m \right)x+k_{y}\left( n \right)y \right]}$$

$E_{z}\left( x,y,z \right)=\sum_{m=-\infty}^{\infty} \sum_{n=-\infty}^{\infty} s_{z}^{m,n}\left( z \right) e^{-j\left[ k_{z}\left( m \right)x+k_{y}\left( n \right)y \right]}$ eq. 2

and

$$H_{x}\left( x,y,z \right)=\sum_{m=-\infty}^{\infty} \sum_{n=-\infty}^{\infty} u_{x}^{m,n}\left( z \right) e^{-j\left[ k_{z}\left( m \right)x+k_{y}\left( n \right)y \right]}$$

$$H_{y}\left( x,y,z \right)=\sum_{m=-\infty}^{\infty} \sum_{n=-\infty}^{\infty} u_{y}^{m,n}\left( z \right) e^{-j\left[ k_{z}\left( m \right)x+k_{y}\left( n \right)y \right]}$$

$$H\left( x,y,z \right)=\sum_{m=-\infty}^{\infty} \sum_{n=-\infty}^{\infty} u_{z}^{m,n}\left( z \right) e^{-j\left[ k_{z}\left( m \right)x+k_{y}\left( n \right)y \right]}$$

where *s* and *u* represent the Fourier series coefficients of the electric field **E** and magnetic field **H**, respectively, at a particular coordinate. Shortly, by substituting the Fourier series expansions into $\boldsymbol{\nabla}\boldsymbol{\times E}=-\frac{\partial\boldsymbol{B}}{\partial t}$ and $\boldsymbol{\nabla}\boldsymbol{\times B}=\mu_{0}\varepsilon_{0}\frac{\partial\boldsymbol{E}}{\partial t}$, the following formula transformations for the TE mode can be obtained after the reduction from the 3D periodic structure case to the 2D grating structure (in an *x-y* plane) and finally to the 1D grating structure (in an *x-y* plane with periodicity along the *x*-axis) [1,2]:

where

$\boldsymbol{K}_{\boldsymbol{i}}=\left( \begin{matrix} k_{i}^{(1,1)} & 0 & 0 \\ 0 & \ddots& 0 \\ 0 & 0 & k_{i}^{(m,n)} \end{matrix} \right)$, $\boldsymbol{u}_{\boldsymbol{i}}=\left( \begin{matrix} u_{i}^{(1,1)} \\ \vdots\\ u_{i}^{(m,n)} \end{matrix} \right)$, and $\boldsymbol{s}_{\boldsymbol{i}}=\left( \begin{matrix} s_{i}^{(1,1)} \\ \vdots\\ s_{i}^{(m,n)} \end{matrix} \right)$

where *i* = *x*, *y,* or *z*.

The wave propagation equation for the TE mode is then given by

$$\frac{d^{2}}{dz}s_{y}-\Omega^{2}s_{y}=0$$

$\Omega^{2}=PQ$, eq. 3

which is a matrix wave equation with

$$P=-\left⟦ 1/\mu_{r} \right⟧^{-1}$$

$Q=\left⟦ \varepsilon_{r} \right⟧-\tilde{K}_{x}\left⟦ \mu_{r} \right⟧^{-1}\tilde{K}_{x}$

On the other hand, the wave propagation for the TM mode can be written as

$$\frac{d^{2}}{dz}u_{y}-\Omega^{2}u_{y}=0$$

$\Omega^{2}=PQ$, eq. 4

which is a matrix wave equation with

$$P=-\left⟦ \frac{1}{\varepsilon_{r}} \right⟧^{-1}$$

$Q=\left⟦ \mu_{r} \right⟧-\tilde{K}_{x}\left⟦ \varepsilon_{r} \right⟧^{-1}\tilde{K}_{x}$.

# References

1. Duraipandian, S., Petersen, J. C., and Lassen,M., Authenticity and Concentration Analysis of Extra Virgin Olive Oil Using Spontaneous Raman Spectroscopy and Multivariate Data Analysis, *Appl. Sci*., 9, 2433 (2019). https://doi.org/10.3390/app9122433 [↑](#endnote-ref-1)
2. Zhao, J. M. & Zhang, Z. M. Journal of Quantitative Spectroscopy & Radiative Transfer Electromagnetic energy storage and power dissipation in nanostructures. *J. Quant. Spectrosc. Radiat. Transf.* **151**, 49–57 (2015). doi: 10.1016/j.jqsrt.2014.09.011 [↑](#endnote-ref-2)
3. Zhao, B. Rigorous Coupled-Wave Analysis (RCWA) MATLAB Code Tutorial. (2014) accessed at http://zhang-nano.gatech.edu/ [↑](#endnote-ref-3)
